# Oriented display of HIV-1 Env trimers by a novel coupling strategy enhances B cell activation and phagocytosis

**DOI:** 10.3389/fimmu.2024.1344346

**Published:** 2024-02-08

**Authors:** Riccardo Di Vincenzo, Jannis Beutel, Philipp Arnold, Yu Wang, Dominik Damm, Pierre Tannig, Anja Lux, Vladimir Temchura, Jutta Eichler, Klaus Überla

**Affiliations:** ^1^ Institute of Clinical and Molecular Virology, University Hospital Erlangen, Friedrich-Alexander-Universität Erlangen-Nürnberg, Erlangen, Germany; ^2^ Department of Chemistry and Pharmacy, Friedrich-Alexander-Universität Erlangen-Nürnberg, Erlangen, Germany; ^3^ Institute of Functional and Clinical Anatomy, Friedrich-Alexander-Universität Erlangen-Nürnberg, Erlangen, Germany; ^4^ Chair of Genetics, Department of Biology, Friedrich-Alexander-Universität Erlangen-Nürnberg, Erlangen, Germany

**Keywords:** HIV-1, antigen display, peptides, neutralizing antibodies, B cell activation, phagocytosis

## Abstract

**Introduction:**

Conformationally stabilized Env trimers have been developed as antigens for the induction of neutralizing antibodies against HIV-1. However, the non-glycosylated immunodominant base of these soluble antigens may compete with the neutralizing antibody response. This has prompted attempts to couple Env trimers to organic or inorganic nanoparticles with the base facing towards the carrier. Such a site-directed coupling could not only occlude the base of the trimer, but also enhance B cell activation by repetitive display.

**Methods:**

To explore the effect of an ordered display of HIV-1 Env on microspheres on the activation of Env-specific B cells we used Bind&Bite, a novel covalent coupling approach for conformationally sensitive antigens based on heterodimeric coiled-coil peptides. By engineering a trimeric HIV-1 Env protein with a basic 21-aa peptide (Peptide K) extension at the C-terminus, we were able to covalently biotinylate the antigen in a site-directed fashion using an acidic complementary peptide (Peptide E) bearing a reactive site and a biotin molecule. This allowed us to load our antigen onto streptavidin beads in an oriented manner.

**Results:**

Microspheres coated with HIV-1 Env through our Bind&Bite system showed i) enhanced binding by conformational anti-HIV Env broadly neutralizing antibodies (bNAbs), ii) reduced binding activity by antibodies directed towards the base of Env, iii) higher Env-specific B cell activation, and iv) were taken-up more efficiently after opsonization compared to beads presenting HIV-1 Env in an undirected orientation.

**Discussion:**

In comparison to site-directed biotinylation via the Avi-tag, Bind&Bite, offers greater flexibility with regard to alternative covalent protein modifications, allowing selective modification of multiple proteins via orthogonal coiled-coil peptide pairs. Thus, the Bind&Bite coupling approach via peptide K and peptide E described in this study offers a valuable tool for nanoparticle vaccine design where surface conjugation of correctly folded antigens is required.

## Introduction

Rational antigen design and the stabilization of viral surface proteins in the prefusion conformation have significantly advanced the elicitation of robust neutralizing antibody responses (1). Immunization with conformationally stabilized soluble Env trimers, mimicking the native-like pre-fusion state of the HIV-1 surface protein, has shown promise in inducing potent neutralizing antibodies (NAbs) against neutralization-resistant (tier-2) HIV viruses in rabbits and similar but weaker responses in macaques (2).

Developing an effective HIV-1 vaccine capable of eliciting broadly neutralizing antibodies has proven to be a challenging endeavor. While such antibodies occasionally emerge following chronic HIV-1 infection, inducing them through vaccination is exceedingly complex (3). One of the prominent obstacles lies in designing an immunogen that consistently presents the gp120-gp41 envelope glycoprotein trimer in its native conformation, mimicking the configuration on the viral surface. Recent advances, exemplified by the SOSIP and NFL Env constructs, have enabled the production of soluble, native-like Env trimers with accurate structural and antigenic attributes (4–7). SOSIP trimers are characterized by the presence of a disulfide bond formed between gp120 and gp41, referred to as sulphur-on-sulphur (SOS), in addition to an isoleucine-to-proline (IP) point mutation at residue 559 (4). Similarly, native flexibly linked (NFL) trimers also feature the I559P mutation present in SOSIP trimers. However, NFL trimers replace the furin cleavage site between the two Env subunits with an extended flexible linker, resulting in trimers that are both covalently linked and cleavage independent (4). These trimeric analogs have demonstrated significant potential by eliciting a neutralizing response against circulating tier-2 viruses. bNAbs typically develop from strain-specific autologous NAbs through a complex interplay involving multiple cycles of viral escape and antibody affinity maturation. More recently, it also became apparent that antibodies encoded by the germ-line sequences of bNAbs did not bind to HIV Env trimers (8–11). Therefore, vaccination with wild type HIV Env trimers may not initiate the affinity maturation process from these germ-line encoded precursors. Major research efforts are thus focused on germ-line targeting strategies based on sequential immunization regimens starting with modified Env proteins bound by the precursor antibodies of bNAbs (10–17). Thus, a practical and versatile display of different Env proteins with precisely defined conformations on the surface of nanoparticle could promote the development of such sequential immunization regimens.

Recent research has also shed light on the immunodominant epitope region located at the base of the BG505 SOSIP.664 trimer (2, 18–22). This region has been found to play a crucial role in eliciting responses from murine B cells following immunization (18). Interestingly, these B cells appear to be drawn to this region since it is less glycosylated compared to the gp120 subunits (18). However, antibodies targeting the trimer base are not expected to neutralize the virus, since access to these epitopes should be sterically blocked by their proximity to the virion membrane (19–21).

One potential solution involves the multivalent presentation of Env trimers on particulate vaccines, effectively concealing the trimer’s base and enhancing the activation of B cells directed toward more relevant epitopes (23–25). This approach holds promise in vaccine design, provided that the conformation of the Env protein is preserved throughout the coupling procedure. Previous studies have indeed demonstrated the feasibility of coating the surface of nanoparticles with HIV-1 Env trimers, leading to increased antigen binding and B cell activation when trimers were presented on calcium phosphate (26) or silica nanoparticles (27). In preclinical investigations, vaccine candidates utilizing engineered protein nanoparticles have demonstrated significant enhancements in the effectiveness and scope of antibody responses against a range of antigens. Examples include prefusion respiratory syncytial virus (RSV) F (28), HIV-1 envelope (29), influenza hemagglutinin (30), and P. falciparum cysteine-rich protective antigen (CyRPA) (31). These improvements were observed in comparison to both soluble antigens and commercially available vaccine benchmarks.

In this investigation, we explored a novel method for the site-selective, cysteine-free chemical modification of proteins, based on covalently stabilized heterodimeric coiled-coil peptides, termed Bind&Bite (32). By engineering a trimeric HIV-1 Env protein to express one of the coiled-coil peptides appended on the C-terminus, we were able to covalently biotinylate the antigen in a site-directed fashion using the complementary coiled-coil peptide bearing a reactive site and a biotin molecule. This ligation technique is based on the creation of isopeptide and squaramide bonds, respectively, between these coiled-coil peptides. Here, we successfully demonstrate the feasibility of this innovative method, utilizing microspheres carriers as a surrogate for nanoparticle vaccines. Our research not only serves as an empirical validation of the Bind&Bite technique but also offers valuable insights into its potential applications. By orchestrating an ordered array of Env trimers, we efficiently address the challenge of diverting the immune response away from the non-glycosylated base of the HIV Env protein, a phenomenon that could distract the immune response from targeting more relevant epitopes.

## Materials and methods

### Env production and purification

The expression plasmid encoding Env-K, a stabilized HIV Env trimer of BG505 NFL2P gp140 (4) with a C-terminal Peptide K (KIAALKEKIAALKEKIAALKE) has been previously described (32). FreeStyle 293F cells were transfected with the Env-K expression plasmids in sterile disposable PETG flasks (Wagner and Munz GmbH, Munich, Germany) with 3 µg polyethylenimine (Sigma Aldrich, Taufkirchen, Germany) per 1 µg DNA. The transfection mix was prepared in OPTI-MEM Reduced Medium (Thermo Fisher, Waltham, MA, USA). Three days post-transfection, supernatants were collected, sterile-filtered through 0.2 µm Minisart filters (Sigma Aldrich, Taufkirchen, Germany), and purified via lectin affinity chromatography with an ÄKTA protein purification system (Cytiva, Marlborough, MA, USA) using agarose-bound lectin from *Galanthus nivalis* (Vector Laboratories Inc., Burlingame, CA, USA), specific to α-1,3- or 1,6- linked D-mannose of carbohydrates. Lectin columns were loaded after washing with PBS containing 1 mM EDTA and 1 mM EGTA (both Sigma Aldrich, Taufkirchen, Germany). After loading, columns were washed again and protein eluted using a 1 M solution of Methyl-α-D-mannopyranoside (Merck, Darmstadt, Germany). Carbohydrates in the eluate were removed by dialysis and the protein was concentrated over Amicon Centrifugal Filters with 30 kDa cut-off (Merck, Darmstadt, Germany). The affinity-purified Env proteins were further purified to size homogeneity using size exclusion chromatography (SEC) on a Sephacryl S-300 column. The trimer fractions were collected and pooled. Protein concentrations were determined using either a bicinchonic acid-based assay (BCA assay; Thermo Scientific, Rockford, IL) or UV_280_ absorbance using the ND100-NanoDrop^®^ (peQlab, Erlangen, Germany).

### SDS-PAGE, blue native-PAGE, and western blot

Purity of Env proteins were analyzed using BN-PAGE or SDS-PAGE and transferred to a nitrocellulose membrane for Western blotting.

BN-PAGE was conducted according to manufacturer’s instructions (Thermo Fisher, Waltham, MA, USA). Briefly, SEC-purified Env-K trimer fractions were pooled together and 4µg were mixed with NativePAGE^®^ Sample Buffer, NativePAGE^®^ 5% G-250 Sample Additive, and deionized H_2_O. 4-16% Bis-Tris pre-cast gels (Thermo Fisher, Waltham, MA, USA) were placed in a gel-running tank and samples and NativeMark™ Unstained Protein Standard (Thermo Fisher, Waltham, MA, USA) were loaded into wells. Cathode buffer chamber was filled with NativePAGE™ Dark Blue Cathode Buffer, whereas the anode buffer chamber with NativePAGE™ Anode Buffer. Gels were run for 1.5 h at 200 V at 4°C.

For SDS-PAGE, samples were mixed at a 1:2 ratio with SDS sample buffer, incubated at 95°C for 5 min and subsequently loaded onto a discontinuous polyacrylamide gel, composed of a 5% stacking and a 10% separation gel. Additionally, 8 µl of PageRuler Protein Ladder (Thermo Fisher, Waltham, MA, USA) was added, in order to determine protein masses. After SDS-PAGE, gels were transferred on a nitrocellulose membrane by semi-dry blotting using Pierce™ 1-Step Transfer Buffer (Thermo Fisher, Waltham, MA, USA). Blotting was followed by blocking of the membrane in 5% skimmed milk for 60 min at RT. Subsequently the membrane was incubated with monoclonal 2G12 antibodies (Polymun, Klosterneuburg, Austria) followed by incubation with a polyclonal goat anti-human IgG HRP (Dianova, Hamburg, Germany). Alternatively, soluble tetrameric streptavidin HRP (Abcam, Cambridge, UK) was used instead of 2G12 to detect covalent biotinylation of Env. Finally, after washing, ECL solution was applied to the membrane and chemiluminescence was detected by the Intas advanced fluorescence imager (Intas, Göttingen, Germany).

### Peptide synthesis

The biotinylated peptide PepE-Bio (Ac-EIAALEREIAALEREIAALER-Aoa-Aoa-Lys(Bio)-NH_2_) was synthesized as an C-terminal amide by Fmoc-based solid phase peptide synthesis, as previously described (32). The Aoa (8-amino-3,6-dioxa-octanoic acid) residues were introduced as a spacer between the peptide sequence and the biotinylated lysine residue (Lys(Bio). The N-terminus was acetylated.

### Biotinylation of Env

Site-directed covalent biotinylation of Env-K was performed using the acidic biotinylated Peptide E (PepE-Bio Ac-EIAALEREIAALEREIAALER-Aoa-Aoa-Lys(Bio)) forming a coiled coil with the peptide K on Env-K, produced in-house as described above.

For Bind&Bite, PepE-Bio was pre-diluted in activation buffer (0.1 M 2-ethanesulfonic acid (MES), 0.5 M NaCl) containing 2 mM 1-Ethyl-3-(3-dimethylaminopropyl)carbodiimide (EDC) and 2 mM N-Hydroxysuccinimide (NHS), resulting in a final PepE-Bio concentration of 200 µM. After a 30-minute incubation period at room temperature, 10 µl of the activated PepE-Bio were added to 90 µl of purified Env-K in a 2-fold molar excess for 1 h at RT, resulting in a final Env concentration of 10 µM.

For non-covalent biotinylation of Env-K (Bind Only), PepE-Bio was first pre-diluted in activation buffer (0.1 M MES 0.5 M NaCl) without EDC/NHS prior to incubation with Env-K in a 2-fold molar excess. The reactions were dialyzed and Env-K biotinylated at the C terminus was concentrated over Amicon Centrifugal Filters with 30 kDa cut-off (Merck, Darmstadt, Germany).

Undirected biotinylation of Env-K was performed using the commercially available kit, Biotinylation Kit/Biotin Conjugation Kit (Fast, Type B) - Lightning-Link^®^ (Abcam, Cambridge, UK), which makes use of primary amine groups. According to manufacturer’s instructions, 100 µg of antigen (Env-K) were incubated with 10 µL of Modifier reagent in order to add sulfhydryl groups at free amines on lysine residues. The modified Env-K was then added to the lyophilized Biotin Conjugation Mix, followed by 15 min incubation at room temperature. 10 µl of Quencher reagent were added to block the reactive groups on any remaining free biotin conjugate label. After an incubation period of at least 5 min, the undirectedly biotinylated Env-K was dialyzed and concentrated over Amicon Centrifugal Filters with 30 kDa cut-off (Merck, Darmstadt, Germany).

### Negative stain electron microscopy

Negative stain transmission electron microscopy (nsTEM) was in principle performed as described before (33, 34). Briefly, biotinylated (after Bind&Bite) and non-biotinylated (before Bind&Bite) samples were diluted to a final concentration of 0.025 mg/ml in 25 mM Tris-HCl 150mM NaCl pH 7.5 buffer, added to a freshly negative glow discharged continuous carbon grid (Science Service Munich, Munich, Germany), and stained with 2% uranyl-acetate solution. Images were acquired on a JEOL1400Plus (JEOL, Freising Germany) at a nominal magnification of 50k, which corresponds to a pixel length of 3.56 Å. Images were transferred to CisTEM (35) for image processing. For non-biotinylated samples 12.763 particles and for biotinylated samples 11.274 particles were included into the final class sums.

### Coating of streptavidin beads with biotinylated Env

Non-fluorescent 1 µm streptavidin-coated polyester magnetic beads (Kisker Biotech, Steinfurt, Germany, PMST-1.0) or yellow-green fluorescent (505/515) 1 µm FluoSpheres™ NeutrAvidin™-Labeled Microspheres (F8776, Invitrogen, Carlsbad, CA, USA) were blocked for 1 h at RT with 3% BSA/PBS, washed twice on a magnetic rack with FACS buffer (0.1% BSA/PBS), and 30.0 x 10^6^ beads were incubated in 200 µl of the different biotinylated Env proteins at a final Env concentration of 20 µg/ml for 1 h at RT on a shaker, after which samples were washed twice with FACS buffer. A ratio of 100 ng of biotinylated Env/1.0 x 10^6^ beads was found to be saturating.

To discriminate between covalent and non-covalent biotinylation of Env-K samples were also incubated with different concentrations of guanidinium chloride (GuHCl) for 20 min at RT. Samples were than extensively washed four times with PBS and stained with Alexa Fluor 647 (AF647)-labelled 2G12 antibodies at a concentration of approximately 1 µg/ml for 30 min at 4°C. Labelling of the 2G12 antibody with AF647 dye was performed using the AF647 Antibody Labelling Kit (Invitrogen, Carlsbad, CA, USA) according to manufacturer’s instructions. Fluorescence intensities of Env coupled beads were assessed via flow cytometry using an Attune NxT instrument (Thermo Fisher, Waltham, MA, USA).

### Quantitative ELISA for Env-coupled beads

Env concentration of Env-coupled beads was determined by quantitative ELISA. For this, 2.0 x 10^7^ Env-coupled beads were diluted 1:1, 1:5, 1:25, 1:125, and 1:625 in coating buffer (15 mM Na_2_CO_3_ + 35 mM NaHCO_3_ in H_2_O, pH 9.6) and two-fold serial dilutions of Env-K (starting at 10 µg/ml) in coating buffer were prepared as Env standard. 100 µl of each sample were coated over night at RT on high-binding 96 well microtiter plates. Using a magnetic plate holder, plates were washed three times with PBS-T, followed by blocking with 3% BSA in PBS-T for 60 min at RT and subsequent incubation with 2G12 antibody (1:5000 in 1% BSA/PBS-T) for 60 min at RT. Alternatively, washes for fluorescent non-magnetic beads were performed by centrifuging the plate at 4000 g for 10 min. After washing the plates three times, HRP-conjugated anti-human IgG (for 2G12 detection) diluted 1:5000 in 1% BSA in PBS-T was added and incubated for 60 min at RT. Finally, plates were washed before addition of 50 µl ECL solution and detection of luminescence in the multilabel plate reader Victor X4 (PerkinElmer, Waltham, MA, USA).

RLU values were used to calculate the concentration of Env present on beads. For this, the RLU values of a negative control lacking the primary antibody were subtracted from all standard samples. The standard curve was then generated, using mean RLUs from the two-fold serial dilution. Background signal was excluded by subtracting the mean RLU value of samples containing uncoated beads. The assessment of absolute Env concentration on beads, displayed as µg/ml values, for each sample was performed by point-to-point analysis in GraphPad Prism 6 software, followed by multiplication with the respective dilution factor of each sample.

### Conformational antibody binding

Env-K was biotinylated and immobilized on magnetic particles as described above. Samples were then stained with monoclonal Env antibodies for 1 h at RT (2G12, Polymun Scientific GmbH, Klosterneuburg, Austria; PGT121, PG9, PGT145, VRC01, 5F3, 240-D, F240, NIH AIDS Reagent Program, Bethesda, MD, USA) at a concentration of 5 µg/ml. Samples were then washed twice with FACS buffer and incubated with AF647-labelled anti-human IgG (Biolegend, San Diego, USA) for 30 min at 4°C. After two additional washes with FACS buffer, samples were analyzed via flow cytometry using an Attune NxT instrument (Thermo Fisher, Waltham, MA, USA).

### B cell activation assay

Untouched resting B cells from PGT121 BCR-transgenic mice (36) were isolated from the spleen by magnetic cell separation with the mouse B Cell Isolation Kit (Miltenyi Biotec, Bergisch Gladbach, Germany). 2.0 × 10^5^ cells were incubated overnight with different concentrations of non-fluorescent magnetic Env-coupled microspheres in U-bottom 96-well plates. Env-concentrations of beads were determined by a quantitative ELISA (as described above) in order to stimulate B cells with same amounts of Env-K biotinylated in an undirected or site-directed manner. Isolated PGT121 B cells were incubated with a 5-fold serial dilution of Env-beads starting at 1 µg Env/ml or with the corresponding number of beads/cells when beads alone were used (uncoated beads), starting at 10 beads/cells. Unstimulated B cells and B cells stimulated with 5 µg/mL of LPS (Sigma-Aldrich, Corp., St. Louis, MO, USA) were used as negative and positive controls, respectively. After 18 h incubation under standard tissue culture conditions, cells were stained with Fixable Viability Dye (Thermo Fisher, Waltham, MA, USA) and with antibodies against the B cell surface antigen CD19-APC (clone 1D3, Biolegend, San Diego, CA, USA), the early activation markers CD69-PerCP/Cy5.5 (clone H1.2F3, BD Biosciences, Franklin Lakes, NJ, USA), CD86-PE/Cy7 (clone GL1, BD Biosciences, Franklin Lakes, NJ, USA), and CD80-FITC (clone 16-10A1, BD Biosciences, Franklin Lakes, NJ, USA). B-cell activation in living B-cells was subsequently measured via flow cytometry using an Attune NxT instrument (Thermo Fisher, Waltham, MA, USA) and analyzed with the FlowJo software (BD Biosciences, Franklin Lakes, NJ, USA).

### Ca^2+^ influx assay

Untouched resting B cells from PGT121 transgenic mice were isolated as described above. B cell activation was assessed using the Fluo-4 Direct™ Calcium Assay Kit (Invitrogen, Carlsbad, CA, USA), according to manufacturer’s instructions. Briefly, isolated B cells were pelleted and resuspended in Fluo-4 Direct™ calcium assay buffer at a density of ~2.5 × 10^6^ cells/mL and incubated for 60 minutes in tissue culture conditions. An equal volume of 2X Fluo-4 Direct™ calcium reagent loading solution was added directly to the cells and samples were incubated for 60 minutes under tissue culture conditions. Following 30 s of baseline measurement, aliquots of 1 x 10^6^ cells/mL were then stimulated for 210 s at RT with 1 µg/ml of Env-coupled beads (normalized on Env concentration) or control reagents. To determine the maximum Ca^2+^ influx, 5 µg/mL ionomycin (Invitrogen, Carlsbad, CA, USA) was used. Fluorescence was measured by an LSR-II flow cytometer (BD Biosciences, Franklin Lakes, NJ, USA) with an excitation at 494 nm and emission at 516 nm. Kinetics analyses were performed using FlowJo v10.8.1 software (BD Biosciences, Franklin Lakes, NJ, USA).

### Phagocytic uptake by THP-1 cells

The assay was performed as described elsewhere with some minor modifications (37). Briefly, 9×10^5^ Env-coupled fluorescent 1 µm neutravidin beads (Invitrogen, Carlsbad, CA, USA) were transferred in each well of a round bottom 96-well plate. After incubation with a 2-fold serial dilution of the antibodies (PG9, PGT121, or 5F3) starting at a concentration of 10 µg/ml for 2 hours at 37°C, immune complexes were washed, and 2×10^4^ THP-1 cells were added to each well and incubated overnight under standard tissue culture conditions in DMEM culture medium supplemented with 10% heat-inactivated FCS, 2mM Glutamine (all from Gibco, Thermo Fisher, Waltham, MA, USA), 1% Penicillin/Streptomycin (complete DMEM medium). The following day, cells were washed and fixed with 2% paraformaldehyde, and plates were analyzed by flow cytometry on an Attune NxT instrument (Thermo Fisher, Waltham, MA, USA). A phagocytic score was calculated by gating on cells using forward and side scatter, and multiplying the percentage of bead-positive cells by the mean fluorescence intensity (MFI) of the bead-positive population. To ensure robust data, a minimum of 2,000 cells per sample were analyzed.

### Animals

Mice transgenic for the PGT121 B cell receptor targeting the HIV-1 surface glycoprotein Env (36) were kindly provided my Michel Nussenzweig for in-house breeding. Mice were accommodated in ventilated cages at the animal facility (Franz-Penzoldt-Center) of the Faculty of Medicine, FAU (Erlangen, Germany) and handled as prescribed by the Federation of European Laboratory Animal Science Association and as approved by the Government of Lower Franconia. Naive animals were euthanized with CO2 in accordance with Section 4 Paragraphs 1 and 3 of the Animal Welfare Act (§4 Abs. 1 und 3 Tierschutzgesetz), registration number: “TS-12/21 Virologie”.

### Statistical analyses

Statistical analyses were performed with GraphPad Prism software 7 (Graphpad Software Inc., San Diego, CA, USA) and significant differences were determined by a one-way ANOVA with Sidak’s multiple comparisons. Significance between each group was considered at: *p<0.05; **p<0.01; ***p<0.001; ****p<0.0001 as indicated in the figure legends.

## Results

### Oriented display of Env trimers by site-directed biotinylation via Bind&Bite

**Figure 1:**
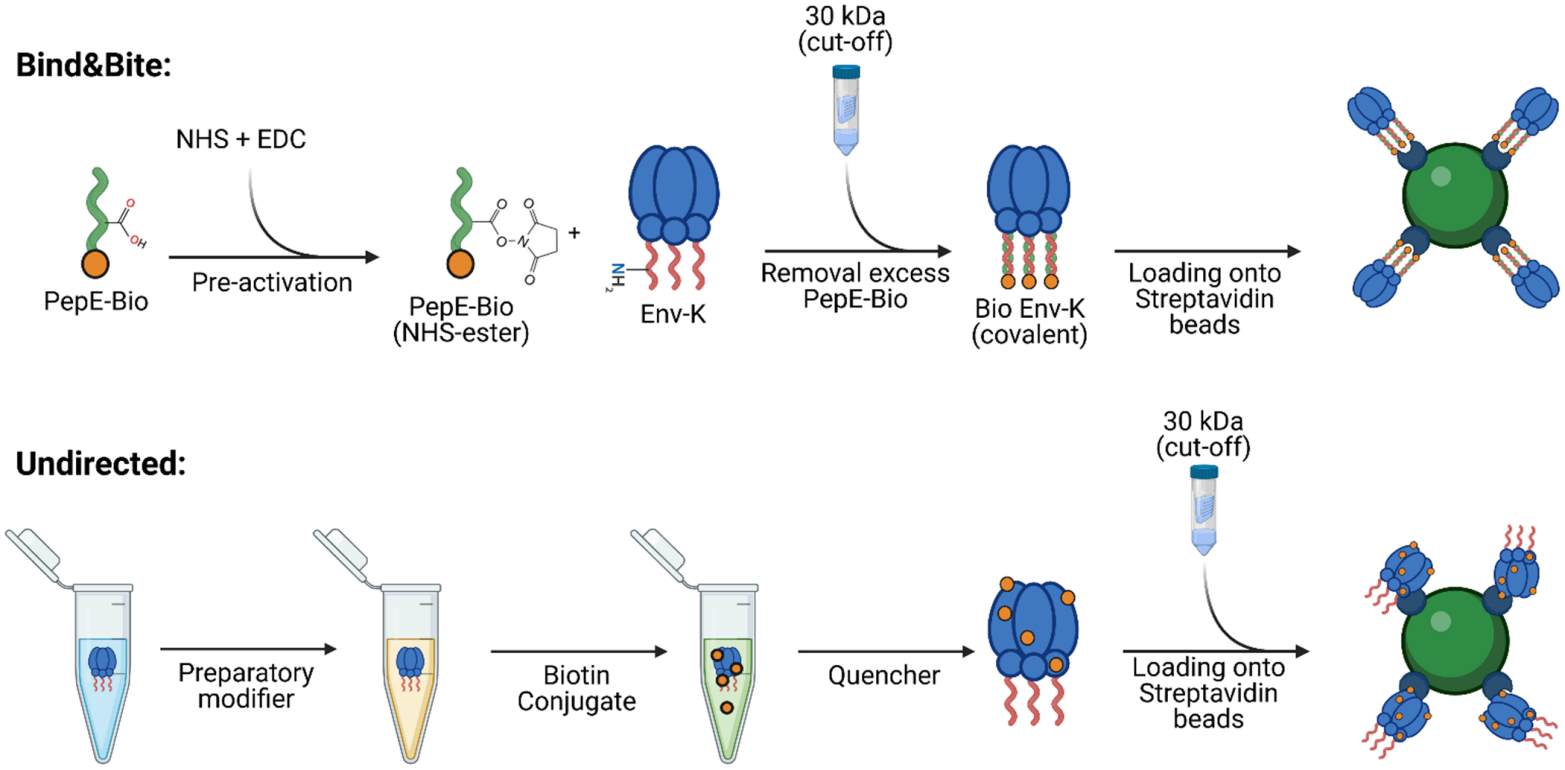
Biotinylation reactions for oriented and undirected display of trimers of HIV Env. To investigate the impact of antigen orientation on IgG recognition, antigen-specific B cell activation, and phagocytic uptake, the same HIV Env protein was biotinylated either at the C-terminus by a recently developed site-selective covalent coupling strategy named “Bind&Bite” (upper panel) or, in an unselective fashion, at free amines (lower panel). The Bind&Bite method involves site-directed modifications of proteins through the formation of heterodimeric coiled coils. An acidic 21 amino acid peptide with a biotin molecule attached to its C-terminus (PepE-Bio) is pre-activated with EDC and NHS, resulting in the formation of NHS esters at glutamate side chains. Subsequently, the activated PepE-Bio is incubated at a 2-fold molar excess with Env-K (HIV-1 BG505 gp140 Env trimer with the complementary Peptide K fused in frame to the C-terminus), followed by the removal of unbound PepE-Bio via dialysis. The purified and concentrated Env-K-Bio is then loaded onto streptavidin-coated beads for further functional analyses. Biotinylation randomly at free amines was performed with a commercial biotin conjugation kit (Abcam). Env-K is treated with the provided “Modifier” reagent to add sulfhydryl groups at free amines. The antigen is then incubated with the biotin conjugate and remaining free labels have their reactive groups blocked via the “Quencher” reagent. Lastly, unbound biotin and buffer is exchanged to PBS through dialysis. PepE-Bio: biotinylated Peptide E; EDC: 1-Ethyl-3-(3-dimethylaminopropyl)carbodiimide; NHS: N-Hydroxysuccinimide. Created with BioRender.com.

In order to compare the effects of an oriented and non-oriented multivalent presentation of antigen on the activation of antigen-specific B cells, we aimed to generate trimeric HIV Env proteins, that are either biotinylated in a site-directed manner at the C-terminus for an oriented display on streptavidin-coated microspheres, or are biotinylated at free amines for a undirected display on the same streptavidin beads (**Figure 1**). Oriented display of soluble antigens on the surface of particles requires site-specific coupling approaches maintaining the conformational integrity of the vaccine antigen. We recently developed such a covalent coupling strategy, designated Bind&Bite, which achieves site-selectivity by covalent ligation of a heterodimeric coiled coil (32). Our antigen of choice, a stabilized HIV Env trimer, was fused to a basic 21-aa peptide (PepK) via a 3xG4S linker resulting in Env-K. PepK can form a coiled-coil with a complementary acidic peptide E (PepE). This charge-driven interaction can be covalently stabilized by the formation of isopeptide bonds between glutamate residues in PepE and lysine residues in PepK (32). If a biotinylated PepE (PepE-Bio) is used for this coupling step, this results in covalent site-directed biotinylation of Env-K (**Figure 1**). Alternatively, incubating PepE-Bio with Env-K without pre-activation with EDC/NHS will result in a non-covalent biotinylation of Env-K, designated Bind Only. To present Env-K in a multivalent, but non-oriented manner, Env-K was also biotinylated at free amines using an amine-based coupling reaction (**Figure 1**).

**Figure 2:**
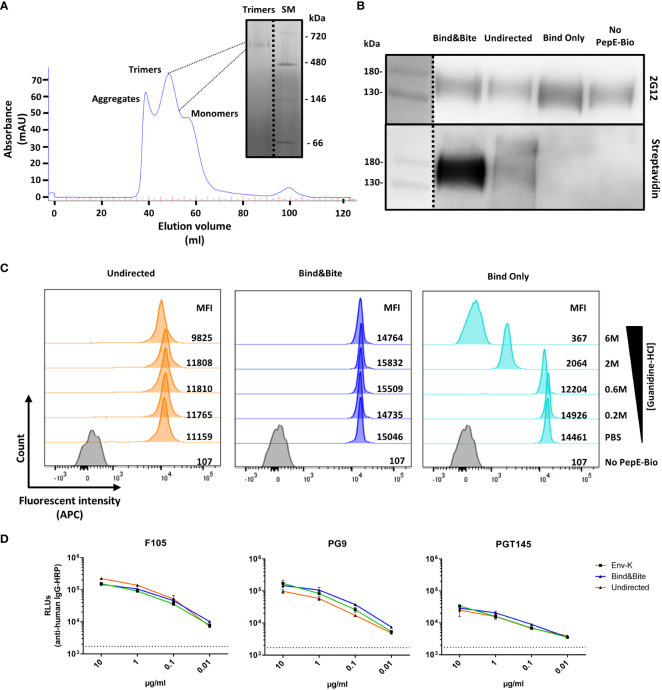
Purification and characterization of biotinylated Env-K. **(A)** Purification of Env-K trimers by SEC chromatography. Peaks corresponding to aggregates, trimers, and monomers as annotated and a blue native (BN)-PAGE of pooled fractions of the trimer are shown. SM: Size marker. **(B)** Western blot analyses of Env-K covalently biotinylated at the C terminus (Bind&Bite) or at free amines (undirected) with 2G12 (upper panel) or streptavidin-HRP (lower panel) under reducing conditions. Non-covalently biotinylated Env-K (Bind Only) and non-biotinylated Env-K (No PepE-Bio) were also analyzed. **(C)** Stability of Env-K-coated streptavidin beads in the presence of the indicated concentrations of guanidinium hydrochloride. MFI: Mean fluorescent intensity. **(D)** ELISA comparing binding of bNAbs for Env-K before (green) and after biotinylation with Bind&Bite (blue) or with the undirected method (orange). Values shown are means ± S.D. of three replicates (n = 3). Dotted black line represents average from three replicates in which secondary Abs were added without bNAbs.

Soluble HIV Env-K, was produced by transient transfection of 293F cells and subsequent purification of the protein via lectin chromatography. HIV Env trimers were then purified by size-exclusion chromatography (SEC) as shown in **Figure 2A**. The purified Env-K trimers were biotinylated either at the C-terminus using biotinylated PepE or by employing a commercially available biotinylation kit. Regardless of the biotinylation method used, HIV Env-K could be detected by Western Blot analysis with the monoclonal antibody 2G12 (**Figure 2B**). To detect the covalent C-terminal biotinylation of Env-K and the undirected biotinylation at free amines distributed over the whole protein, a second Western Blot was stained with streptavidin-coupled peroxidase. While covalently biotinylated Env K and Env K biotinylated at free amines were readily detectable (**Figure 2B**), non-covalently biotinylated Env K was not. Most likely, this is due to dissociation of non-covalently bound biotinylated PepE from Env-K upon exposure to the denaturing conditions of the SDS Page preceding the Western blot analyses (**Figure 2B**).

**Figure 3:**
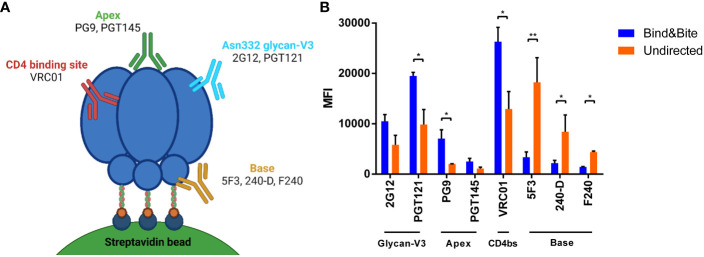
Accessibility of epitopes of mAbs targeting HIV Env. **(A)** Schematic view of binding sites of the indicated mAbs on the HIV Env trimer. Created with BioRender.com. **(B)** Binding of the indicated antibodies to beads coated with Env-K in an oriented or undirected manner. Means and standard deviations of the MFI of triplicate FACS measurements from one representative of two independent experiments are shown. Statistical analysis was performed using a one-way ANOVA with Sidak’s multiple comparisons. *p<0.05; **p<0.01.

Binding of Env-K protein biotinylated by the different methods to streptavidin-coupled microspheres was then analyzed under saturating Env-K concentrations by flow cytometry using the fluorescently labelled 2G12 antibody. Covalent and non-covalent biotinylation at the C-terminus of Env-K resulted in a slightly higher mean fluorescence intensity of the microspheres than amine-based biotinylation of Env-K, possibly due to better accessibility of Env (as shown later in **Figure 3**). The mean fluorescence intensity of microspheres coated with Env was examined by exposing them to increasing concentrations of guanidinium hydrochloride, a chaotropic agent known for disrupting the non-covalent interactions responsible for maintaining the native conformation of a protein (38). This treatment resulted in a significant reduction in the MFI of microspheres displaying Env-K that was non-covalently coupled. In contrast, the binding of Env-K that was either biotinylated at the C-terminus or attached via free amines was only marginally affected by this treatment. (**Figure 2C**). To assess if Bind&Bite alters the conformational integrity of the Env protein, we conducted an ELISA using the mAbs F105 (binding to the open conformation of trimers) and PG9 and PGT145 (both binding to the closed conformation of trimers). Although the Env trimer used in our study exhibited a partially open conformation even before labelling, our results demonstrated no significant difference in antibody binding before and after employing the Bind&Bite approach (**Figure 2D**). This was further confirmed by nsEM data (**Supplementary Figure S1**). From samples prior and post Bind&Bite approach ~12.000 single particles were submitted to image processing to calculate class sum images. In both data sets the frequency of similar class sums was high. The typical C3 symmetric “top view” was observed equally often (**Supplementary Figure S1**, red circles). Additionally, we identified slight asymmetric class sums in both data sets, representing most likely Env trimers with one monomer in an open conformation (**Supplementary Figure S1**, white arrows). Our results indicate that the Bind&Bite method does not adversely affect the trimeric integrity, supporting the validity of our approach for multimerization.

### Accessibility of epitopes

To explore whether the different coupling strategies affect the accessibility of different epitopes of Env-K, binding of a panel of broadly-neutralizing monoclonal antibodies to Env-K presented on microspheres was compared by flow cytometry. Our panel included two antibodies targeting V3 glycans (2G12, PGT121), one bNAb against the CD4-binding site (VRC01), two broadly neutralizing antibodies specifically directed against conformational epitopes situated at the apex of the Env trimer (PG9, PGT145), and three against the trimer base (5F3, 240-D, F240). Our results revealed enhanced binding by the two monoclonal antibodies targeting V3 glycans and the two bNAbs against the apex of the Env trimer when Env-K was presented on microspheres through site-directed coupling at the C-terminus (**Figure 3B**). In contrast, when Env-K was coupled to microspheres in an undirected manner, all base antibodies displayed stronger staining (**Figure 3B**). These observations suggest that the oriented coupling of Env-K via its C-terminus enhances the accessibility of epitopes associated with various classes of broadly-neutralizing monoclonal antibodies, while simultaneously concealing epitopes located at the base of the trimer.

### B cell activation

**Figure 4:**
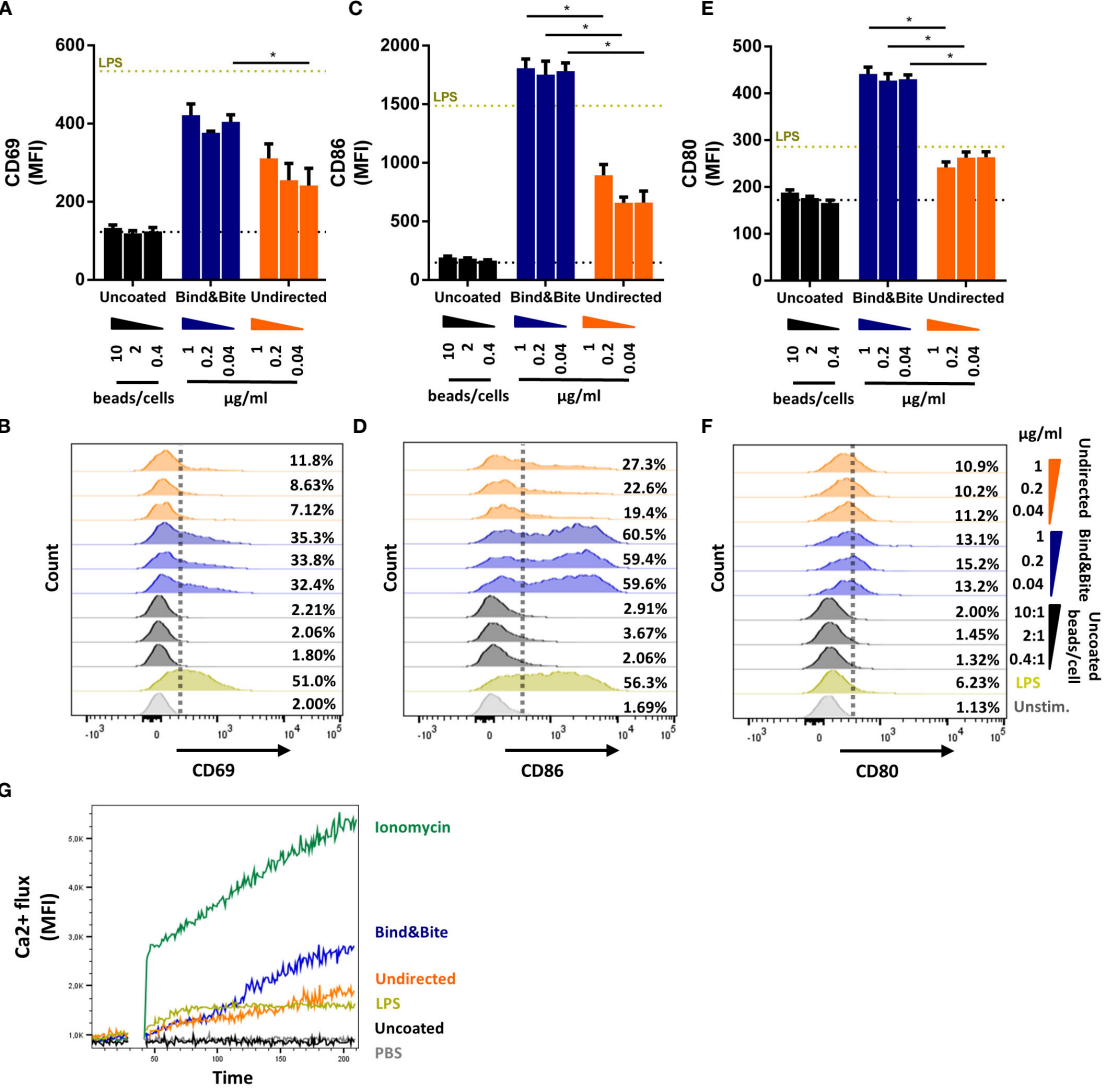
B-cell activation. B cells isolated from splenocytes of PGT121 transgenic mice were incubated with beads coated with Env-K biotinylated either with Bind&Bite (blue) or in an undirected (orange) manner. Uncoated beads were used as negative control. Expression of CD69 **(A, B)**, CD86 **(C, D)** and CD80 **(E, F)** activation markers after overnight incubation with the indicated concentrations of Env or ratios of uncoupled beads to cells. Expression levels for unstimulated cells (dotted black line) and cells stimulated with 5 µg/ml LPS (dotted yellow line) are also indicated. Means and standard deviations of the MFI of triplicate FACS measurements from one representative of two independent experiments are shown. **(G)** Activation of PGT121 B cells as measured by Ca2+ flux over 210 seconds after stimulation with beads displaying Env-K biotinylated either with Bind&Bite (blue) or in an undirected (orange) manner, with ionomycin (green) or LPS (yellow). Uncoupled beads (black) and PBS only (grey) were also included to determine nonspecific activation. Data from one representative of two independent experiments are shown. Statistical analysis was performed using a one-way ANOVA with Sidak’s multiple comparisons. *p<0.05.

We also compared Env-specific PGT121 B cell activation in the presence of equal amounts of Env trimers that were immobilized onto beads either in an oriented or undirected manner *in vitro*. As a control, we assessed the impact of uncoupled beads lacking the Env trimers (termed “uncoated”) in this assay. After titrating varying amounts of Env on a fixed number of beads, we could find a ratio at which all beads were fully saturated with biotinylated Env-K, which was equivalent to around 100 ng/1.0 x 10^6^ beads for both undirected and oriented labelling methods (data not shown). To ascertain that both coupling strategies resulted in beads with an equivalent amount of Env, the Env concentration of the beads was quantified by ELISA (**Supplementary Figure S2**). The number of uncoated particles used in the controls for stimulating PGT121 B cells was matched with the number of Env coupled beads used. This revealed that when Env trimers were presented on microspheres in an oriented manner, an enhanced expression of B cell activation markers could be detected. Specifically, CD69, CD86, and CD80 were upregulated following an overnight incubation when compared to B cell activation by Env trimers coupled in an undirected manner (**Figures 4A–F**). The enhanced activation of PGT121 B cells by microspheres presenting Env in an oriented manner was stable over a wide range of beads/cell ratios excluding minor differences in Env content or bead numbers as the underlying cause.

To determine whether this heightened B cell activation resulted from improved cross-linking of the B cell receptor through oriented antigen presentation, we also looked for early events in the B cell activation pathway, particularly the mobilization of intracellular calcium. Our findings indicated that stimulation with beads after site-directed C-terminal coupling of HIV Env led to an increased mobilization of intracellular calcium within minutes (**Figure 4G**). This observation suggests that site-directed coupling of the antigen can facilitate more efficient cross-linking of the PGT121 B cell receptor, as it was reflected by enhanced intracellular calcium mobilization, in comparison to the presentation in an undirected manner.

### Phagocytosis

**Figure 5:**
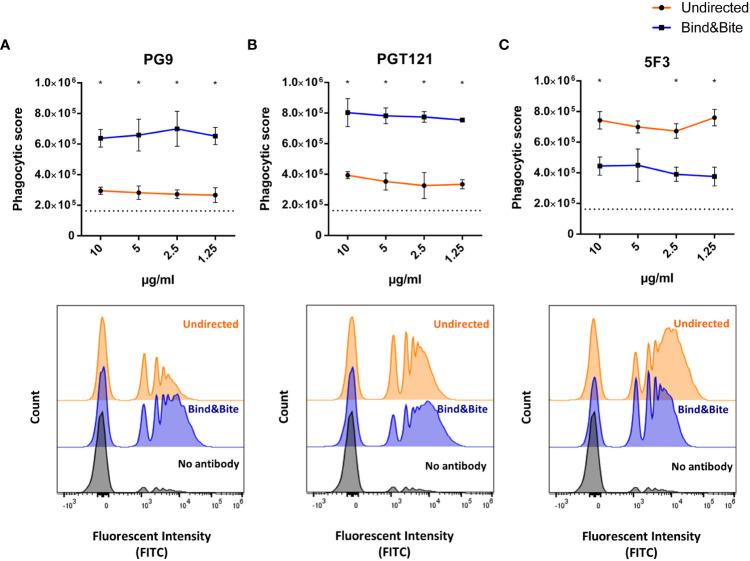
Phagocytosis. Fluorescently labelled beads displaying Env-K biotinylated either with Bind&Bite (blue) or in an undirected (orange) manner were opsonized with saturating concentrations of the mAbs PG9 **(A)**, PGT121 **(B)** or 5F3 **(C)** prior to incubation with THP-1 monocytic cells. Uptake of the beads by the THP-1 cells was determined by flow cytometry and the phagocytosis score was calculated as the percentage of bead-positive cells × MFI of bead-positive cells. Mean and standard deviations of triplicates from one representative of two independent experiments are shown. Non-opsonized beads were included as controls (dotted black line). Representative histogram of the flow cytometric analyses is shown for beads opsonized with the indicated antibodies at a concentration of 2.5 µg/ml. Statistical analyses were performed using a one-way ANOVA with Sidak’s multiple comparisons. *p<0.05.

Finally, we investigated the impact of the oriented antigen display on phagocytic uptake, using the THP-1 monocytic cell line as a model system. The amount of biotinylated Env per bead was empirically calculated as described above and kept equivalent for beads displaying Env in an oriented and undirected manner. Overnight incubation of THP-1 cells with equal number of Env-coupled microspheres opsonized with PG9 or PGT121 mAbs revealed superior uptake of beads when antigen was displayed in an oriented manner. Conversely, incubation with the base-binding 5F3 antibody resulted in a reduced phagocytic score for the oriented antigen display with respect to the undirected presentation (**Figure 5**). These results are consistent with our earlier findings, indicating that oriented antigen immobilization improves the display of neutralizing epitopes of Env, while also concealing non-neutralizing ones located at the base of the protein. Furthermore, this improved antigenicity is associated with more efficient uptake by phagocytic cells.

## Discussion

B-cell targeting nanoparticle-based vaccines require the precise immobilization of properly folded antigens onto their surfaces to enhance immunogenicity. In this investigation, we explored a novel method for the covalent modification of antigens, termed Bind&Bite (32), to enable their oriented attachment to a carrier. Specifically, we established the feasibility of this approach using microspheres as carrier. Our study not only provides empirical validation of this coupling approach but also sheds light on its potential applications. By orchestrating an ordered array of Env trimers, we could potentially address the challenge of distraction of the immune response to the immunodominant epitopes found at the non-glycosylated base of the HIV Env protein, as could be shown by Bale et al. for maleimide-based antigen-coupling onto liposomes (24).

We directly compared the Bind&Bite based labelling approach to a commercially available non-targeted conjugation kit. Both demonstrated efficient biotinylation of the antigen. However, a stronger signal in the western blot analysis was observed when staining with streptavidin-HRP for Env-K that was covalently biotinylated using our Bind&Bite system, compared to the sample biotinylated at free amines. This is unexpected because the latter method should theoretically introduce more biotin molecules per Env trimer, resulting in a stronger signal. One possible explanation for this observation could be related to the biotin conjugation kit used in the study, the Biotin Conjugation Kit (Fast, Type B) - Lightning-Link^®^ from Abcam. This kit is specifically optimized for producing conjugates intended for assays in which the biotinylated protein is captured by streptavidin immobilized on a surface (e.g., plates, nitrocellulose, magnetic beads, etc.). In contrast, the company suggests using the Type A Biotin Conjugation Kit to produce conjugates for assays in which a streptavidin-labelled detection reagent will be employed. However, our choice to use the Type B kit was based on the investigative purpose of our study, which aimed to compare the effect of different methods of protein immobilization on microspheres.

Nevertheless, the covalent biotinylation of the HIV Env-K trimer, confirmed by resistance to guanidinium chloride treatment, and the successful coupling onto streptavidin-coated beads validate the feasibility and reliability of our site-directed labelling system. Moreover, our study demonstrated no significant difference in binding of conformational antibodies before and after employing the Bind&Bite approach, therefore indicating that our labelling method does not adversely affect the trimeric integrity, supporting the validity of our approach for multimerization. It is, however, important to notice that the Env trimer used in this study exhibited partial openness pre-labelling, which could be partially due to the use of lectin instead of PGT145 columns for purification. Despite this being the case, it is crucial to clarify that the primary focus of our study is not the employment of the “perfect” native-like trimer but rather to ascertain that the Bind&Bite itself does not alter the Env trimer’s conformation and to demonstrate the feasibility of its practical application.

Although an enzymatic site-directed biotinylation is also possible using the Avi-tag (39), it should be noted that the Bind&Bite method can be readily adapted to alternative chemical reactions for the covalent modification of proteins, which also enables the selective modification of two or more proteins, using orthogonal pairs of coiled-coil peptides as adapter molecules (32). If the biotin of PepE is replaced with a reactive group used in click chemistry, such as dibenzocyclooctyne (DBCO) or maleimide, Bind&Bite could also be applied for other carrier systems, providing a versatile tool for antigen display in vaccine design.

Through antibody binding assays using a panel of bNAbs targeting different epitopes, we investigated the structural integrity of our biotinylated Env-K trimers displayed on microspheres and the potential enhancement of binding resulting from an oriented antigen display. Our results demonstrated augmented binding of desirable neutralizing antibodies and reduced binding to epitopes located at the base of the Env protein. It is worth noting that the enhanced binding of neutralizing antibodies observed with our labelling system supports the hypothesis that an oriented array of Env trimers promotes the exposure of neutralizing epitopes while minimizing accessibility of the ones located at the base of the protein.

The assessment of B cell activation in our study has disclosed that oriented Env trimers, when presented on microspheres, stimulate a substantial elevation in the expression of B cell activation markers when compared with undirected antigen presentation. This result suggests that oriented antigen presentation fosters a more efficient induction of B cell activation, conceivably through an augmented cross-linking of B cell receptors. This hypothesis is further supported by the observed increase in intracellular calcium mobilization presented in this study as well as by three other studies. Damm et al. (26) demonstrated that calcium phosphate nanoparticles (CaPs) displaying orthogonally arranged Env trimers on their surface (o-CaPs) were superior in activation of Env-specific B-cells (*in vitro*) and induction of Env-specific antibody responses (*in vivo*) compared to CaPs with Env trimers coupled in a randomly oriented manner. In a second study, Thalhauser et al. (40) showed that the uptake of Env attached to silica nanoparticles (SiNPs) via a site-specific covalent conjugation was 4.5-fold enhanced, whereas adsorbed Env resulted only in a moderate 1.4-fold increase compared with Env in its soluble form. In a similar manner, Peterhoff et al. (41) demonstrated that an oriented, covalent germline-targeting (GT) HIV Env conjugation revealed better binding of structure-dependent bNAbs as compared to non-specifically adsorbed GT-Env. In addition, GT-Env covalently attached activated a B cell line expressing the germline VRC01 receptor at an EC_50_ value in the nanomolar range, while soluble GT-Env required 1,000-fold higher concentrations to induce signaling. B cell activation was induced irrespective of GT-Env attachment mode even if the kinetics and extent of activation was different.

I53-50, a self-assembling NP vaccine platform, utilizes a prefusion trimer-stabilized SOSIP envelope antigen fused to a trimeric secondary building block (29, 42). Displaying ConM SOSIP on I53-50 significantly boosts neutralizing antibody titers compared to SOSIP alone. Conversely, BG505 SOSIP on I53-50 results in lower titers and reduced neutralizing response compared to BG505 SOSIP alone, attributed to restricted accessibility to BG505’s immunodominant epitopes at the spike’s base. This feature of SOSIP-I53-50NPs, concealing the trimer base, has potential for enhancing NAb-lineage activation. Moreover, SOSIP-I53-50NPs shape vaccine-induced responses by reducing Ab responses to the gp120-gp41 interface, valuable for strategies aiming at triggering apex-specific Ab responses.

Our observations with the Bind&Bite approach seamlessly align with the findings from I53-50 (29, 42) and other nanoparticle delivery systems (26, 40, 41), strongly suggesting an important role for antigen orientation in influencing immune responses, particularly in B cell activation.

Furthermore, we investigated how the orientation of antigens affects their uptake by phagocytic cells, using the THP-1 monocytic cell line as a model. Our findings revealed that when antigens were presented in a specific orientation, there was a significant increase in the uptake of beads coupled with the Env antigen, especially when exposed to bNAbs like PG9 or PGT121. Conversely, when the antigen was displayed in an undirected manner, the likelihood of exposing epitopes located at the base of the antigen was higher. This, in turn, led to an increased uptake by phagocytic cells following opsonization with antibodies that target the base. These results indicate that opsonized beads displaying antigens in a specific orientation are more efficiently taken up. Similar to more efficient stimulation of the BCR, the opsonization of Env displayed in an ordered manner by a monoclonal antibody may also lead to more efficient activation of Fc-γ receptors mediating the Fc dependent phagocytosis.

Although we did not yet address the induction of antibody responses *in vivo*, B cell activation and phagocytic uptake are early events of the immune response and can be considered surrogate markers of immunogenicity. Further investigations, including assessments of immune responses in animal models, are warranted to comprehensively evaluate the potential of the oriented antigen display approach for integration into the domain of nanoparticle vaccine development. However, the studies mentioned earlier (26, 29, 41, 42) show promising results in support of this hypothesis.

The advantages offered by our Bind&Bite approach are therefore three-fold: i) the preservation of protein conformation throughout the coupling procedure; ii) the ability to present an oriented array of Env trimers displayed on a microsphere carrier, potentially overcoming the limitations associated with biasing immune responses towards undesired epitopes; iii) compared to other coupling strategies, the adaptability of the complementary PepE to be customized with alternative chemical reactions for the covalent modification of proteins, therefore expanding its applicability beyond the sole particle surface conjugation with antigens.

In conclusion, our study provides evidence that the use of an oriented array of Env trimers displayed on a microsphere carrier enhances B cell activation, improves antigenicity, and promotes the accessibility of neutralizing epitopes. The Bind&Bite approach for covalent modification of antigens described here offers a valuable tool for antigen display in nanoparticle vaccine design in general.

## Data availability statement

The original contributions presented in the study are included in the article/**Supplementary Material**. Further inquiries can be directed to the corresponding authors.

## Ethics statement

Ethical approval was not required for the studies on humans in accordance with the local legislation and institutional requirements because only commercially available established cell lines were used. The animal study was approved by the governmental authorities of Lower-Franconia under the license number TS-12/21 Virologie. The study was conducted in accordance with the local legislation and institutional requirements.

## Author contributions

RV: Conceptualization, Data curation, Formal analysis, Investigation, Methodology, Visualization, Writing – original draft. JB: Writing – review & editing, Investigation, Methodology. PA: Investigation, Writing – review & editing, Methodology. YW: Investigation, Writing – review & editing. DD: Investigation, Writing – review & editing. PT: Investigation, Writing – review & editing. AL: Methodology, Supervision, Writing – review & editing. VT: Supervision, Writing – review & editing. JE: Conceptualization, Funding acquisition, Resources, Supervision, Writing – review & editing. KÜ: Conceptualization, Funding acquisition, Methodology, Project administration, Resources, Supervision, Writing – original draft, Writing – review & editing.
