# Supplementary material for: Oriented display of HIV-1 Env trimers by a novel coupling strategy enhances B cell activation and phagocytosis
Source: Front Immunol. 2024 Feb 8;15:1344346. doi: 10.3389/fimmu.2024.1344346 (PMC10882061; doi:10.3389/fimmu.2024.1344346)
Supplement: Supplementary file 1 [file DataSheet_1.docx]

Supplementary Material

## Supplementary Figure 1 (S1)


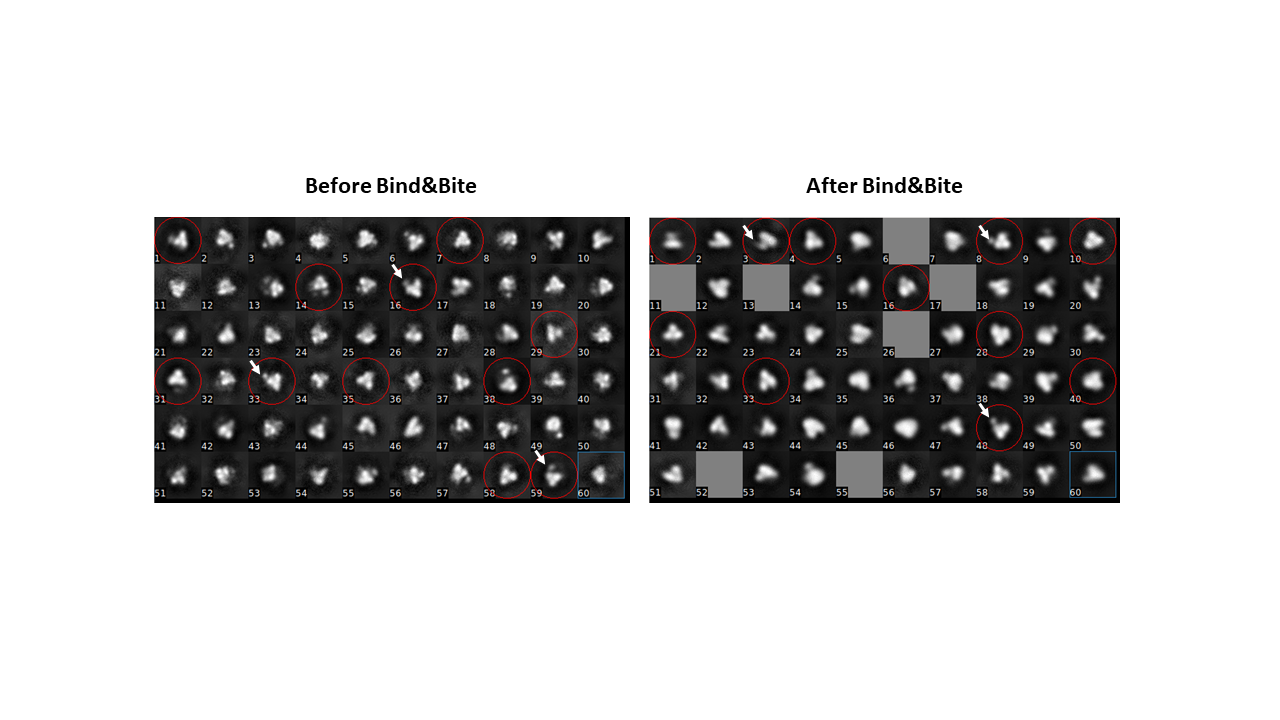


**Figure S1: nsEM stain before and after Bind&Bite.** Negative stain EM data of pre- and post-biotinylation using Bind&Bite of Env-K trimers that were first affinity-purified with lectin columns and then by SEC with a Sephacryl S-300 column. Shown are class sum images. The top views (typical c3 symmetry) are marked with red circles, whereas slight asymmetries are indicated with white arrows. Both sets have very symmetric class sums in closed conformation (e.g. Before Bind&Bite: 31, 33, 58; After Bind&Bite: 10, 28, 33) and class sums with a slight asymmetry (e.g. Before Bind&Bite: 1, 16, 59; After Bind&Bite: 8, 48), likely due to one monomer in open conformation.

## Supplementary Figure 2 (S2)





Figure S2: Quantitative ELISA of Env-coupled beads. To measure the amount of biotinylated Env-K present on streptavidin-coated microspheres, we performed a titration of beads coated with Env-K in an oriented (blue) or undirected (orange) manner and with uncoated beads (black) as a control. (A) Binding to the recombinant proteins was checked with a fixed concentration of 2G12 mAb. (B) Env concentration on beads from Point-to-point analysis. Values shown are means ± s.d. of two replicates (n = 2).
